# Pseudolaric Acid B Targets CD147 to Selectively Kill Acute Myeloid Leukemia Cells

**DOI:** 10.3390/ijms25126517

**Published:** 2024-06-13

**Authors:** Sheng Zou, Ekaterina Parfenova, Nikolina Vrdoljak, Mark D. Minden, Paul A. Spagnuolo

**Affiliations:** 1 Department of Food Science, University of Guelph, Guelph, ON N1G 2W1, Canada; szou02@uoguelph.ca (S.Z.); eparfeno@uoguelph.ca (E.P.);; 2 Princess Margaret Cancer Center, Ontario Cancer Institute, Toronto, ON M5G 2M9, Canada; mark.minden@uhn.ca

**Keywords:** pseudolaric acid B (PAB), CD147 (EMMPRIN/BSG/BASIGIN), acute myeloid leukemia (AML), nuclear factor kB (NF-κB) pathway, cell proliferation, apoptosis

## Abstract

Acute myeloid leukemia (AML) is an aggressive blood cancer. With low survival rates, new drug targets are needed to improve treatment regimens and patient outcomes. Pseudolaric acid B (PAB) is a plant-derived bioactive compound predicted to interact with cluster of differentiation 147 (CD147/BSG). CD147 is a transmembrane glycoprotein overexpressed in various malignancies with suggested roles in regulating cancer cell survival, proliferation, invasion, and apoptosis. However, the detailed function of PAB in AML remains unknown. In this study, AML cell lines and patient-derived cells were used to show that PAB selectively targeted AML (IC50: 1.59 ± 0.47 µM). Moreover, proliferation assays, flow cytometry, and immunoblotting confirmed that PAB targeting of CD147 resulted in AML cell apoptosis. Indeed, the genetic silencing of CD147 significantly suppressed AML cell growth and attenuated PAB activity. Overall, PAB imparts anti-AML activity through transmembrane glycoprotein CD147.

## 1. Introduction

Acute myeloid leukemia (AML) is an aggressive hematological malignancy originating in the bone marrow that is characterized by uncontrolled proliferation and accumulation of abnormal blast cells that fail to develop into functional blood cells [1,2]. The 5-year net survival rate of 21% among patients aged 15 to 99 years [3] and the dose-limiting toxicities of current regimens [4,5,6,7,8] highlight the glaring need for improved drugs and drug targets. 

Through an unbiased screen, pseudolaric acid B (PAB) was identified as a potential anti-AML compound. It is a diterpene acid found in the root bark of the *Pseudolarix kaempferi* Gordan tree (Golden Larch) [9,10] with noted anti-fungal, anti-cancer, anti-inflammatory, and anti-angiogenetic properties [11,12,13,14,15]. PAB induces apoptosis in leukemia cell lines [16,17,18] and targets cluster of differentiation (CD) 147, as identified using chemical proteomics [19]. 

CD147, also known as Basigin (BSG) or extracellular matrix metalloproteinase inducer (EMMPRIN), is emerging as a potential anti-cancer therapeutic target [20,21,22,23]. CD147 stimulates multiple signaling pathways and regulates proteins such as nuclear factor kB (NF-κB), B-cell lymphoma 2 (Bcl-2) family, and matrix metalloproteinases (MMPs) [15,24,25,26,27,28,29,30]. Notably, CD147 is overexpressed in AML cells (primary patient and patient-derived cell lines), compared to normal CD34+ hematopoietic progenitor cells [22]. In other cell types, overexpressed CD147 upregulates downstream NF-κB and anti-apoptotic Bcl-2 pathways, thereby inhibiting apoptosis and enabling cancer cell survival [23,24,25,26,31,32]. 

AC73 is a specific inhibitor of CD147. It has been reported to inhibit CD147 dimerization and prevent migration of hepatocarcinoma cells in vitro and in vivo [33]. Mechanistically, it suppresses the ERK/STAT3 activation pathway and imparts cell death by downregulating CD147. However, to significantly inhibit cell proliferation, high doses of AC73 are needed [22,34], which will limit its potential clinical utility. In this study, we evaluated the anti-leukemia effects of PAB, showed that PAB suppressed downstream pathways of CD147 to induce apoptosis of AML cells, and confirmed the role of CD147 in AML proliferation. 

## 2. Results

### 2.1. PAB Exhibits Anti-Leukemia Activities In Vitro

**Figure 1:**
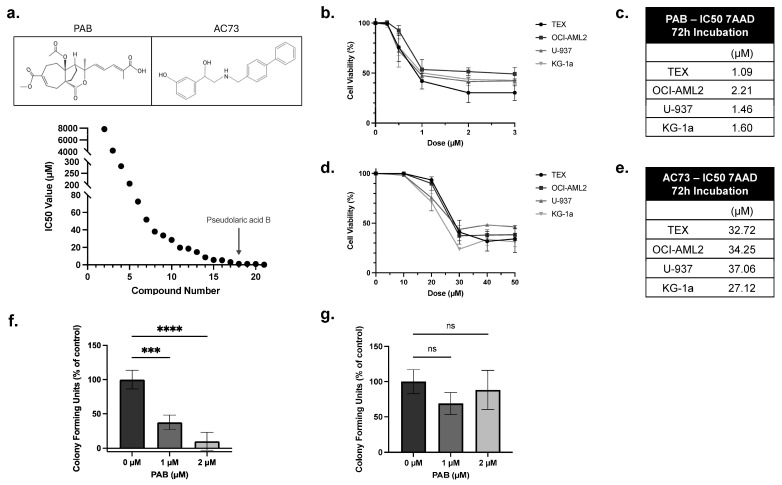
PAB is a potent anti-leukemia compound. (**a**) A drug screen identified PAB as a potent anti-AML drug compound. (**a**, **Top**) PAB and AC73 structure. Leukemia cell lines were treated with increasing concentrations of (**b**) PAB or (**d**) AC73. After 72 h, cell viability was measured by flow cytometry using 7-AAD staining. IC50 values for (**c**) PAB and (**e**) AC73 were calculated from the dose responses in (**b**,**d**). Values were the average of three replicates performed in triplicate (n = 3). (**f**) Primary patient-derived human AML mononuclear cells were incubated with 1 or 2 µM PAB, and colonies were counted after 14 days (n = 4). (**g**) Peripheral blood stem cells (PBSCs) from healthy donors were incubated with 1 or 2 µM PAB, and colonies were counted after 14 days (n = 4). *** *p* ≤ 0.001, **** *p* ≤ 0.0001. Unpaired, one-way ANOVA paired with a Dunnett’s multiple comparisons test was used.

A drug screen of an in-house library identified PAB as a potent anti-AML compound (Figure 1a). To validate this finding, PAB dose-response curves were generated using a panel of leukemia cell lines. Here, cells were treated with PAB or AC73 at increasing concentrations, and after 72 h, viability was measured using 7-AAD staining (Figure 1b,d). PAB lowered leukemia cell viability at low micromolar concentrations (IC50 value: 1.59 ± 0.47 µM) (Figure 1c). In contrast, the known CD147 inhibitor, AC73, exhibited weak anti-leukemia properties (IC50 value: 32.79 ± 4.18 µM) (Figure 1e). 

To evaluate selective activity, PAB was tested in normal and patient-derived AML mononuclear cells using colony formation assays. Primary AML colonies were significantly reduced by 60 or 90%, compared to the control, when treated with 1 µM or 2 µM of PAB, respectively (Figure 1f, F_2,9_ = 54.24, *p* < 0.0001). In contrast, no significant effects were noted in normal PBSC cells at equivalent concentrations (Figure 1g). These results show that PAB is a potent and selective anti-AML compound. 

### 2.2. PAB Regulates CD147, NF-κB, and Bcl-2 Expression in AML Cell Lines

**Figure 2:**
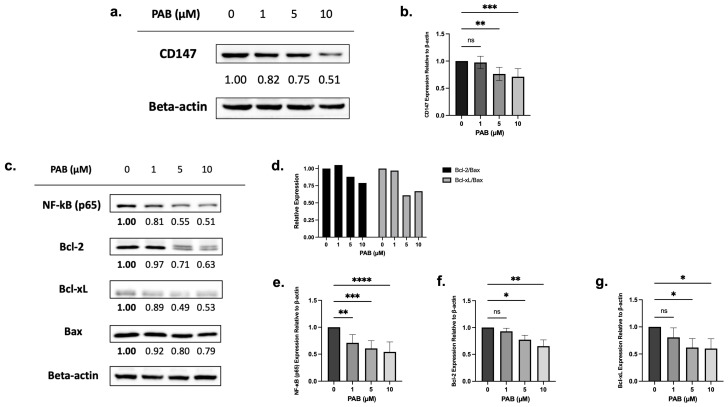
The effects of 24 h PAB treatment on CD147, NF-κB (p65), Bcl-2, and Bcl-xL expression on AML2 cells. (**a**) AML2 cells were treated with 1, 5, or 10 µM PAB for 24 h and effects on CD147 protein levels were assessed by immunoblotting. Representative blots are shown. (**b**) CD147 protein levels were quantified by densitometry and expressed as arbitrary units (AU) by normalization to beta-actin (n = 7). (**c**) AML2 cells were treated with 1, 5, and 10 µM PAB for 24 h and protein levels of NF-κB (p65), Bcl-2, and Bcl-xL were assessed by immunoblotting. Representative blots are shown. (**d**) Densitometry (expressed as AU) for Bcl-2 and Bcl-xL relative to Bax: Bcl-2/Bax and Bcl-xL/Bax ratios were calculated. (**e**–**g**) Densitometry analysis (n = 3) for downstream signal targets in (**c**). * *p* ≤ 0.05, ** *p* ≤ 0.01, *** *p* ≤ 0.001, **** *p* ≤ 0.0001 (n.s. = not significant). Unpaired, one-way ANOVA paired with a Dunnett’s multiple comparisons test.

Chemical proteomics, followed by testing in HeLa cells, showed that PAB targets and reduces CD147 expression [19]. To test the effects on CD147 expression, leukemia cells were treated with PAB at doses and times preceding cell death, and protein levels were measured by immunoblotting. PAB, at increasing concentrations, significantly reduced CD147 protein levels in AML cells (Figure 2a,b, F_3,20_ = 10.97, *p* < 0.001; Supplementary Figure S1a,b, F_3,7_ = 11.19, *p* < 0.01). 

Next, immunoblotting was used to examine the relationship between CD147 protein levels and downstream targets (e.g., NF-κB, anti-apoptotic Bcl-2 and Bcl-xL, and pro-apoptotic Bax). Before cell death, PAB significantly reduced NF-κB (p65) (F_3, 19_ = 12.72, *p* < 0.0001), Bcl-2 (F_3,7_ = 11.04, *p* < 0.01), and Bcl-xL (F_3,8_ = 4.668, *p* < 0.05) expression, which also led to lowered Bcl-2/Bax and Bcl-xL/Bax ratios (Figure 2c–g; Supplementary Figure S1c,d). These findings suggest that PAB can regulate NF-κB, Bcl-2, Bcl-xL, and Bax in a dose-dependent manner. 

### 2.3. PAB Induces Apoptosis in AML Cells

**Figure 3:**
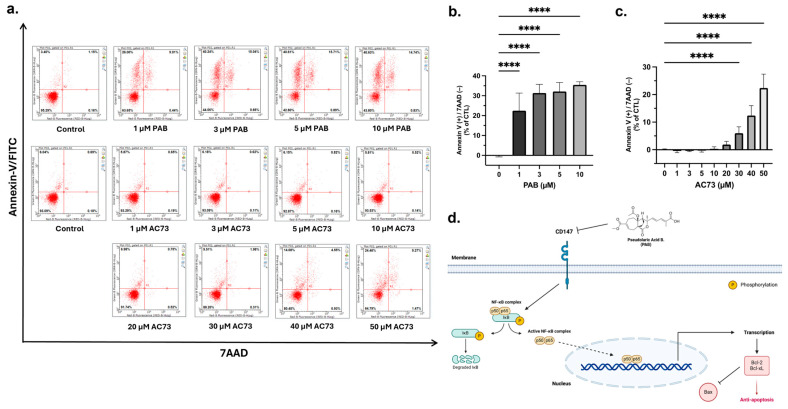
PAB induces apoptosis in AML2 cells. (**a**–**c**) PAB and AC73-treated AML2 cells were incubated for 24 h (i.e., before cell death), and apoptosis was analyzed by flow cytometry using Annexin-V FITC/7AAD staining (n = 3 for PAB treatment, n = 5 for AC73 treatment). **** *p* ≤ 0.0001. Unpaired, one-way ANOVA and a Dunnett’s multiple comparisons test were used. (**d**) Schematic outline of PAB-induced CD147-mediated cell death.

Overexpression of CD147 is associated with the inhibition of cancer cell apoptosis [23,32,35]. Therefore, our observation that PAB reduces CD147 protein levels (Figure 2a,b) led to the hypothesis that PAB decreases CD147 to cause cancer cell apoptosis; a notion supported by several studies [16,18,36]. To determine the effects in AML, apoptosis was analyzed by flow cytometry using Annexin-V FITC/7AAD staining (Figure 3a–c). PAB induced AML cell apoptosis, as noted by a significant increase in the ANN^+^/7AAD^-^ cell population (F_4,42_ = 76.98, *p* < 0.0001). In comparison, AC73 did not induce significant apoptotic effects until reaching 30 µM (F_8,90_ = 155.9, *p* < 0.0001). Indeed, nearly 50 µM of AC73 was required to induce apoptotic effects comparable to that of 1 µM PAB. These findings strongly suggest that PAB can induce apoptosis in AML cells at a relatively low concentration by targeting CD147. A schematic of PAB targeting CD147 is presented in Figure 3d.

### 2.4. Overexpressed in AML Cells, CD147 Regulates AML Cell Growth and Alters NF-κB and Bcl-2 Family Protein Expression

**Figure 4:**
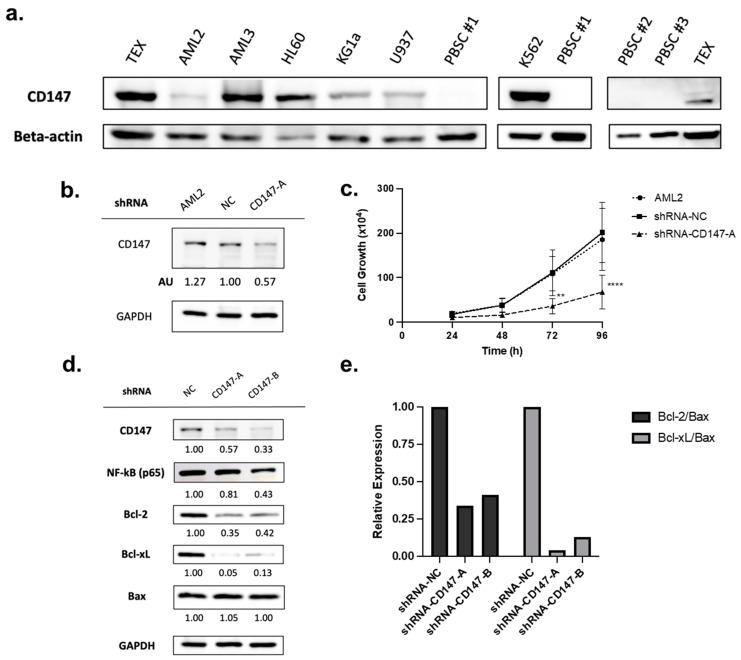
CD147 is highly expressed in leukemia cells but not in normal peripheral blood stem cells (PBSCs) and knockdown impairs growth. (**a**) Immunoblotting for CD147 protein levels in leukemia cell lines and normal PBSCs. (**b**) AML2 cells were transfected with scrambled shRNA (negative control, NC) or shRNA-CD147-A (i.e., targeting CD147). CD147 knockdown was validated by immunoblotting (densitometry (AU) values shown below the blot). (**c**) Proliferation assay on AML2 cells transfected with shRNA-NC or shRNA-CD147-A. Cell growth was assessed using trypan blue. Knockdown of CD147 in AML2 cells significantly restrained cell growth after 72 h (n = 3). ** *p* ≤ 0.01, **** *p* ≤ 0.0001. Unpaired, two-way ANOVA paired with a Dunnett’s multiple comparisons test. (**d**) AML2 cells were transfected with scrambled shRNA (NC), shRNA-CD147-A, or shRNA-CD147-B. The effects of CD147 knockdown on NF-κB (p65), Bcl-2, and Bcl-xL expression were evaluated by immunoblotting analysis (n = 3). Representative blots are shown. (**e**) Relative expressions of Bcl-2 and Bcl-xL relative to Bax: Bcl-2/Bax and Bcl-xL/Bax ratios based on densitometry. Relative expression ratios for the representative blots are shown.

CD147 is overexpressed in cancer cells [25,32], including leukemia cell lines and patient-derived leukemia blasts (as compared to CD34+ hematopoietic progenitor cells) [22]. To validate protein level expression, a panel of leukemia cell lines was tested and compared to normal peripheral blood stem cells (PBSCs). CD147 was highly expressed in all AML cell lines tested, while no expression was detected in normal PBSCs (Figure 4a). 

Overexpression of CD147 has been linked to cell survival and proliferation in cancer cell lines [32,37]. To evaluate the role of CD147 in AML cell proliferation, shRNA-mediated CD147 knockdown (transfected with shRNA-NC or shRNA-CD147-A) was conducted. Knockdown was confirmed by immunoblotting (Figure 4b), and proliferation assays showed that leukemia cells lacking CD147 had significantly reduced growth rates (Figure 4c, *p* < 0.0001). 

PAB reduced CD147 expression (Figure 2a,b) leading to the suppression of the NF-κB pathway and altered expression of Bcl-2 proteins favoring apoptosis (Figure 2c–g and Figure 3a–c). To determine if CD147 knockdown recapitulates the effects of PAB, the immunoblotting of AML2 cells transfected with shRNA-NC, CD147-A, or CD147-B was conducted. Genetic suppression of CD147 in AML2 cells led to reduced endogenous protein levels of NF-κB (p65), Bcl-2, and Bcl-xL (Figure 4d). There was no change in Bax expression; therefore, lower Bcl-2/Bax and Bcl-xL/Bax ratios were noted (Figure 4e). Together, these results confirm that reductions in CD147 result in cellular effects favoring apoptosis and align with observations from the PAB treatment model. 

### 2.5. Knockdown of CD147 Reduces PAB-Mediated Cytotoxicity in AML Cells

**Figure 5:**
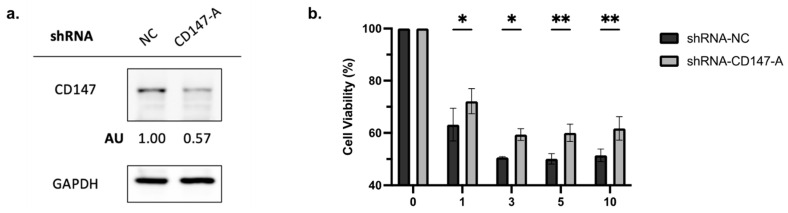
Knockdown of CD147 in AML2 cells results in a decrease in PAB-mediated cytotoxicity. (**a**) AML2 cells were transfected with scrambled shRNA (NC) or shRNA-CD147-A. CD147 knockdown was confirmed by immunoblotting analysis. (**b**) AML2 cells were transfected with shRNA-NC or shRNA-CD147-A. Cell viability was assessed using flow cytometry with 7AAD staining after 48 h incubation (n = 3). * *p* ≤ 0.05, ** *p* ≤ 0.01. Unpaired, two-way ANOVA with a Šidák test.

To elucidate the role of CD147 in PAB-induced cytotoxicity, AML2 cells were transfected with shRNA-NC or shRNA-CD147-A. Knockdown of CD147 was confirmed by immunoblotting (Figure 5a). The viability of PAB-treated, CD147-knockdown AML2 cells was measured after a 48 h incubation by 7AAD staining. PAB-mediated cytotoxicity was significantly reduced in CD147-knockdown AML2 cells (Figure 5b, *p* < 0.0001). These findings indicate that CD147 plays a crucial role in mediating the cytotoxic effects of PAB in AML cells. 

## 3. Discussion

AML is a devastating disease requiring novel therapeutic approaches. In this study, PAB was identified as a molecule capable of inducing selective AML cell toxicity in vitro. Mechanistically, targeting the surface transmembrane protein CD147 resulted in changes in apoptotic proteins favoring leukemia cell death. 

CD147 overexpression is associated with cancer cell proliferation and survival, and leukemia cells have elevated levels compared to normal CD34+ hematopoietic progenitor cells [22,25,32]. Overexpression of CD147 leads to the dysregulation of the NF-κB pathway in cancer cells [26,30,38], primarily through the degradation of the inhibitor of κB (IκB), which allows for the activation and nuclear translation of p50/p65 dimers [39]. Once in the nucleus, active NF-κB dimers can promote the transcription of genes that contribute to the malignant phenotype [40,41,42,43,44]. Specifically, NF-κB can upregulate anti-apoptotic Bcl-2 proteins (e.g., Bcl-2 and Bcl-xL) [31,45,46,47], which in turn suppress pro-apoptotic proteins (e.g., Bax and Bak), thereby promoting cell survival and proliferation [48,49,50]. Consequently, the inhibition of CD147 suppresses the NF-κB pathway and alters anti- and pro-apoptotic protein ratios to favor apoptosis induction. In this study and consistent with previous observations, CD147 was highly expressed in AML but not in normal PBSCs. This differential expression confirms that CD147 could serve as a unique molecular target in AML. Further, the knockdown of CD147 in AML cells suppressed growth and proliferation, reduced protein levels of NF-κB (p65), Bcl-2, Bcl-xL, and lowered ratios of Bcl-2/Bax and Bcl-xL/Bax. These findings confirm the link between CD147 and the regulation of downstream targets (i.e., NF-κB and Bcl-2 family proteins) in AML, suggesting the role of CD147 in signaling pathways that contribute to disease pathophysiology. 

The role of CD147 in malignancies is emerging and several studies point toward the anti-cancer role of reducing CD147 [25,32,51,52,53,54]. There are, however, few pharmacological agents that target this key transmembrane protein. AC73 [3-{2-[([1,1′-biphenyl]-4-ylmethyl) amino]-1-hydroxyethyl} phenol] inhibits the motility and invasion of hepatocellular carcinoma cells [33], and induces anti-proliferative effects in leukemia cells [22]. However, high IC50 values (32.79 ± 4.18 µM) would significantly limit its clinical use. PAB was identified as a possible CD147 inhibitor using a chemical proteomics approach and was confirmed to bind to CD147 in HeLa cells [19]. PAB, a diterpene acid found in *Pseudolarix kaempferi* [10], is used in Traditional Chinese Medicine for uses that include the killing of cancer cells [11,12,13,14,15]. PAB has also been shown to inhibit cancer cell growth, induce cell cycle arrest, initiate ferroptosis, and trigger apoptosis or autophagy. It kills cancer cells of various solid tumor origin, including liver, esophageal, brain, and colon [15,36,55,56,57,58]. Similarly, its action on hematological cancer cell lines has been noted (i.e., KBM5, K562, and U937 cells) [16,18,36]. Flow cytometry results from this study show, for the first time to our knowledge, that PAB significantly induced apoptosis at relatively low doses in AML cell lines and patient-derived AML cells. 

PAB had a low IC50 (1.59 ± 0.47 µM) and induced apoptosis by targeting CD147. Indeed, PAB-treated cells had a phenotype resembling CD147 knockdown cells. The pharmacological inhibition, or genetic knockdown, of CD147 reduced AML cell growth and decreased levels of NF-kB and ratios of Bcl-2/Bax and Bcl-xL/Bax. Finally, leukemia cells with decreased CD147 expression were less sensitive to PAB, further supporting the role of CD147 in PAB-induced death. While these results point toward a unique mechanism of PAB-induced selective AML cell apoptosis, the detailed molecular mechanism by which PAB targets CD147 requires further investigation. In addition, the bioavailability of PAB, which would significantly impact its therapeutic potential and optimal dosing strategies, requires characterization. Finally, the absence of in vivo studies limits our understanding of PAB’s pharmacodynamics and is a focus of current research. 

In summary, this study demonstrated the potent in vitro anti-leukemia activity of PAB, a plant-derived bioactive compound, against AML by specifically targeting and inhibiting CD147, a transmembrane protein of the immunoglobulin superfamily, leading to the suppression of CD147’s downstream anti-apoptotic pathways and induction of apoptosis in AML cells. 

## 4. Materials and Methods

### 4.1. Cell Culture

Several leukemia cell lines (OCI-AML2 (AML2), TEX, KG-1a, U-937, HL-60, and K562) were used. AML2 and KG-1a cells were cultured in IMDM (Iscove’s modified Dulbecco’s medium; WISENT Inc.; Saint-Jean-Baptiste, QC, Canada) supplemented with 10% fetal bovine serum (FBS), 200 units/mL penicillin, and 200 units/mL streptomycin. TEX cells were cultured in IMDM, with 20% FBS, 2 mM L-glutamine (Thermo Fisher Scientific; Waltham, MA, USA), 100 units/mL penicillin, 100 mg/mL of streptomycin, 20 ng/mL stem cell factor (SCF), and 2 ng/mL interleukin-3 (IL-3; Peprotech; Cranbury, NJ, USA). U-937 cells were maintained in RPMI 1640 medium (WISENT Inc.), with 10% FBS, 100 units/mL penicillin, and 100 mg/mL of streptomycin. 

Cell lines were grown in T25 or T75 vented filter cap tissue culture flasks (Sarstedt; Nümbrecht, Germany) and incubated in 5% CO_2_ at 37 °C.

### 4.2. Compounds

AC73 (3-{2-[([1,1′-biphenyl]-4-ylmethyl) amino]-1-hydroxyethyl}phenol) (CAS Number: 775294-71-8; MedChemExpress; Monmouth Junction, NJ, USA) and pseudolaric acid B (PAB) ((3R,4S,4aS,9aR)-4a-(acetyloxy)-3-[(1E,3E)-4-carboxy-1,3-pentadien-1-yl]-3,4,4a,5,6,9-hexahydro-3-methyl-1-oxo-1H-4,9a-ethanocyclohepta[c]pyran-7-carboxylic acid, 7-methyl ester) (CAS Number: 82508-31-4; Cayman Chemical; Ann Arbor, MI, USA) were dissolved in 20% dimethyl sulfoxide (DMSO) (Sigma-Aldrich, St. Louis, MO, USA) and diluted in the corresponding cell culture media, with a final DMSO concentration of no more than 0.5%. 

### 4.3. Cell Viability Assay 

Cell viability was measured using the 7-aminoactinomycin D exclusion assay (7-AAD; Cayman Chemicals). Leukemia cells were seeded in triplicate on a 96-well plate at a concentration of 1.25–5 × 10^5^ cells/mL (in 95 µL fresh media). Cells were treated with increasing concentrations of PAB or AC73 (5 µL of each drug to produce the desired final concentration). The remaining wells were filled with 100 μL of phosphate-buffer saline (PBS). After 48 or 72 h, cells were harvested by centrifugation at 1200 rpm for 5 min, resuspended in 250 µL of 1 µg/mL 7-AAD, diluted in PBS and analyzed using a flow cytometer (Guava EasyCyte 8HT; EMD Millipore; Burlington, MA, USA), as previously described [59]. 

### 4.4. Apoptosis Assay

Cell viability and apoptosis were measured using Annexin-V FITC/7-AAD exclusion. Leukemia cells were seeded in 96-well plates (1.25–5 × 10^5^ cells/mL) and treated with increasing doses of PAB or AC73 for 24 h. Following treatment, cells were collected and resuspended in 50 μL of staining solution consisting of an Annexin V binding buffer (Biovision; Exton, PA, USA) with Annexin V-FITC (150 μg/mL; Biovision) and incubated in the dark for 15 min at room temperature. The cells were then washed with 100 µL Annexin V binding buffer, centrifuged at 1200 rpm for 5 min, and incubated with 7AAD (1 µg/mL; Cayman Chemicals) for 5 min. Following incubation, fluorescence was detected by flow cytometry (Guava^®^ easyCyteTM 8HT; EMD Millipore) as previously described [60].

### 4.5. Colony Formation Assays

Colony formation assays were performed with primary cells obtained with written informed consent provided by Princess Margaret Cancer Centre (Toronto, ON, Canada). Cells were stored in 10%DMSO, 50% FCS, and alpha-MEM at −150 °C. Primary cells (i.e., patient-derived human AML mononuclear cells or normal peripheral blood stem cells (PBSCs) from healthy donors) were collected according to approved human ethics protocols. Cells were suspended in MyeloCult^TM^ H5100 media (StemCell Technologies; Vancouver, BC, Canada). In a 15 mL tube, 300 μL of cell suspension was added to 3 mL of MethoCult GF H4435-enriched methylcellulose medium (StemCell Technologies). The cells were plated in a 35 mm cell culture dish (Corning; Tewksbury, MA, USA) with 1 × 10^5^ cells/dish for patient-derived AML cells or 1 × 10^4^ cells/dish for normal PBSCs using a 5 mL syringe with a blunt cannula (Covodein; Minneapolis, MN, USA); increasing doses of PAB (0, 1, and 2 µM) were added. Duplicates of each treatment were incubated in 100 mm cell culture dishes (Corning) with an additional uncapped 35 mm dish containing distilled water to control the humidity. The plates were incubated for 7–14 days at 37 °C with 5% CO_2_ and 95% relative humidity. The colonies were counted on an inverted microscope; clusters of 50 or more cells were counted as one colony.

### 4.6. Retroviral Mediated Knockdown of CD147 

CD147 gene-specific short hairpin RNA (shRNA-CD147-A and shRNA-CD147-B; OriGene; Rockville, MD, USA; see Supplementary Table S1 for sequences) and a scrambled shRNA negative control (shRNA-NC; OriGene) were transfected into HEK293T cells (cultured in antibiotic-free DMEM media with 10% FBS). Transfection was carried out with a TransIT-LT1 transfection reagent (MirusBio; Madison, WI, USA), according to the manufacturer’s protocol. The cells were cultured at 37 °C after transfection. At 48–72 h post-transfection, the media was collected from the culture and centrifuged at 2000× *g* for 5 min to remove the cell debris. The supernatant was used as viral stock for further transduction. 

For transduction, 1 mL of viral stock and 100 µg/mL of protamine sulfate were added to AML2 cells. At 24 h post-transduction, virus-containing media was removed and replaced with fresh IMDM media containing 0.9 µg/mL of puromycin to select for infected AML2 cells. The selection pressure was maintained for 1–2 weeks, and the media was replaced as needed. The knockdown efficiency was measured by immunoblotting. 

### 4.7. Immunoblot Analysis

Cells were collected and lysed using radioimmunoprecipitation assay (RIPA) buffer (Sigma-Aldrich). The bicinchoninic acid (BCA) protein assay was utilized to quantify the concentration of protein present within the sample. Using equal quantities of protein (20–30 µg), samples were boiled in sodium dodecyl sulfate (SDS) at 95 °C for 5 min before loading onto a 10% SDS-polyacrylamide gel. Proteins were separated using electrophoresis at 150 V for one hour. Transfer of proteins from the gel to a polyvinylidene difluoride (PVDF) membrane was conducted at 25 V for 10 min. The membrane was then blocked with a solution of 5% skim milk or bovine serum albumin (BSA) in tris-buffered saline Tween (TBS-T) for one hour. The membrane was incubated with a CD147 (JF1-045) (1:2000; Thermo-Fisher Scientific), NF-κB p65 (D14E12) (1:1000; Cell Signaling Technology; Danvers, MA, USA), Bcl-2 (D17C4) (1:1000; Cell Signaling Technology; Danvers, MA, USA), Bcl-xL (54H6) (1:1000; Cell Signaling Technology), or Bax (1:1000; Cell Signaling Technology) primary antibody at 4 °C overnight; the loading control was GAPDH or beta-actin (1:4000; ProteinTech; Rosemont, IL, USA). Following incubation, membranes were rinsed with TBS-T and incubated with an appropriate secondary antibody (1:10,000; Abcam; Cambridge, UK) for one hour. After the membrane was rinsed with TBS-T, enhanced chemiluminescence solution (Bio-Rad; Hercules, CA, USA) was added and incubated for 0.5 to 2 min. Membranes were then imaged with Azure 280 Imaging System (Dublin, CA, USA) for protein visualization, and ImageJ 1.54 software was used for densitometric analysis. 

### 4.8. Statistical Analysis

Statistics were evaluated using GraphPad Prism 9.0 software (GraphPad Software, La Jolla, CA, USA). Unless otherwise indicated, the results are expressed as a mean ± the standard deviation. IC50 best-fit values (µmol/L) were analyzed by nonlinear regression (curve fit) and where appropriate, analysis was carried out using a one-way or two-way ANOVA with a Dunnett’s multiple comparisons test, or a two-way ANOVA with a Šidák test. Differences between values were deemed significant if *p* ≤ 0.05. 

## Data Availability

Data available upon request.

## Supplementary Materials

The following supporting information can be downloaded at: https://www.mdpi.com/article/10.3390/ijms25126517/s1.
