# Supplementary material for: Pseudolaric Acid B Targets CD147 to Selectively Kill Acute Myeloid Leukemia Cells
Source: Int J Mol Sci. 2024 Jun 13;25(12):6517. doi: 10.3390/ijms25126517 (PMC11203802; doi:10.3390/ijms25126517)
Supplement: Supplementary file 1 [file ijms-25-06517-s001.zip › ijms-3022037-supplementary.pdf]

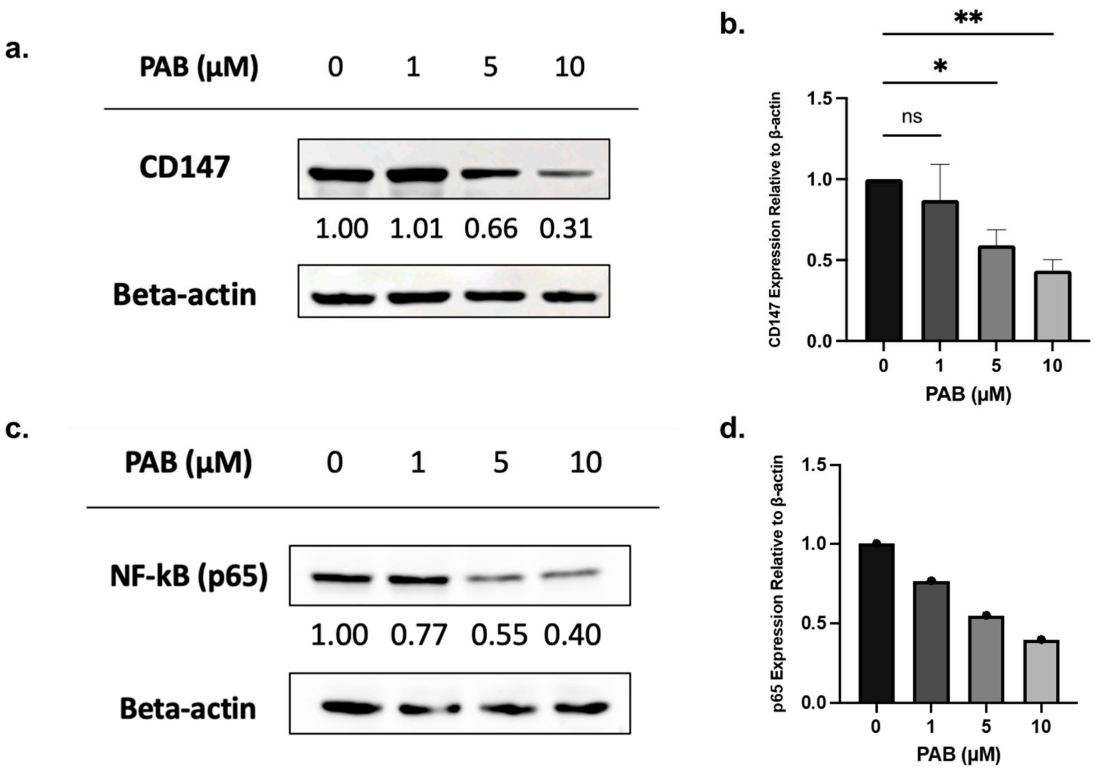

Figure S1. PAB effects on CD147 and NF-κB expression in TEX cells. TEX cells were treated with 1, 5, and 10 µM PAB for 24 h. The effects of PAB treatment on CD147 and NF-κB expression in TEX cells were assessed by immunoblotting. The representative blots are shown. * $p \leq 0.05$, ** $p \leq 0.01$ (n.s. = not significant).

Table S1. CD147 shRNA Sequences.

	**CD147 shRNA Sequence**
**shRNA-CD147-A**	CTTCACTACCGTAGAAGACCTTGGCTCCA
**shRNA-CD147-B**	CAGCACCAGAATGACAAAGGCAAGAACGT
**shRNA-NC**	GCACTACCAGAGCTAACTCAGATAGTACT
